# American Plans for Dealing with War Neuroses

**Published:** 1918-08

**Authors:** 


**Outline of American Plans for Dealing with War Neuroses.** Lt.-Colonel Salmon, M. R. C., Senior Consultant in Neurology for the A. E. F., said that he was sure that all American medical officers are profoundly grateful for the opportunity to hear at first hand the experiences of their French and British colleagues. Their observations, based upon opinions matured by four years of thoughtful work, are invaluable at this time.

In speaking briefly of some American plans for dealing with the medical and military problems created by the extraordinary prevalence of the war neuroses, the speaker said he desired to emphasize the fact that these plans are, in the main, only adaptations for our own needs of all that has seemed most valuable in the work of our allies.

*General Features of American Plans.* American officers are under no illusions as to the importance of psychoneuroses in armies in the field nor as to the difficulties to be encountered in managing certain phases of their treatment and control. In this, as in other work of the Medical Department of the A. E. F., the Chief Surgeon regards reduction in the wastage of men as an issue of paramount importance. No one realizes more keenly than he that in the final analysis not ships, nor food, nor anything else will win the war but men — effective fighting men behind bayonets. It is realized, too, that the problems in this field which will soon confront us in almost as large a measure as they have our colleagues must be approached with an open mind not burdened with preconceptions or over-valued ideas. Some of the factors go so deeply into the currents of human life that we are certain to court failure if we attempt to solve them by any rules of thumb or by applying any rigidly formulated administrative plan. We must utilize all the resources that are provided by neurology and psychiatry and add to them a generous portion of common sense.

As the speaker had viewed these problems — in the light of experience — there have seemed to be three outstanding requirements which must be met as a preliminary to successful attack upon them.

1. A rational attitude toward these disorders on the part of the medical officers, line officers, enlisted men, and the public.

2. A careful sifting of the human material that is to be brought across the Atlantic and fed into the furnace of war.

3. Determination that all which is undertaken from the trenches to the sea shall have for its primary object not the proof, or disproof, of some theory, but the restoration, or conservation, of fighting men for the line.

As to the first requirement we must already admit partial failure. Efforts have been made at home to provide accurate descriptions of the war neuroses. Part of the activities of the Medical Officers' Training Camps has been illustrated by lectures on what "shell shock" is and what it isn't. The cable censorship, upon the request of the Surgeon General's Office, has eliminated the term "shell shock" from despatches from overseas. The term has also been eliminated from the official nomenclature of diseases in use in the A. E. F. In spite of these efforts, however, it seems to be on everybody's tongue. The best that can be hoped for is to sow seeds of distrust as to its fitness or propriety. Nevertheless, efforts are being continued to inform not only medical officers but line officers as to the real nature of war neuroses.

Much more success has been attained in sifting the Army that is now coming into the trenches. This undertaking has been carried on upon a large scale and with carefully planned methods.

In attaining the third of the objects that we have set before us, the necessity of placing the management of the very earliest phases of our problem in expert hands seemed uppermost. We have been profoundly impressed by the experience of the British and French in which certain exigencies of hospital evacuation made it almost impossible at times to keep these cases out of the wards of general hospitals. We have seen, in even our limited experience, these disorders pass from very fluid states into hardest neuroses in the wards of general hospitals.

Our chief reliance in preventing this misadventure is upon the provision of divisional psychiatrists for all tactical divisions. These medical officers are carefully selected with reference to their training and personality. They are not medical consultants to the divisions to which they are attached but just as really members of the divisional sanitary personnel as the officers serving in Field Hospitals, Ambulance Companies, or Regiments. It is their duty to deal with all these cases at the earliest possible moment and it is believed that it will be practicable to prevent the evacuation to hospitals in the S. O. S. of men with neuroses except upon the divisional psychiatrists.

Already a considerable degree of success has attended this plan. In addition to these duties, the divisional psychiatrists are able to give the division surgeons much assistance in dealing with the psychiatric phases of such problems as delinquency, self-inflicted wounds, etc. Several medical officers serving as divisional psychiatrists are here and it is hoped that they will speak in the discussion of their experiences.

As many beds as are required and can be spared are set aside in one divisional field hospital in each division for these cases. Here work of the greatest value to the Army is carried on and the larger part of the men received in these wards from the trenches are sent back within a week or ten days.

The next provision for dealing with the neuroses is the special hospital — Base Hospital No 117 — in the Zone of Advance. In this hospital all the therapeutic measures found most useful elsewhere are provided.

It is realized, however, that this hospital will be relatively of little service if the cases of war neuroses admitted have already been transformed into " ward neuroses " in the general hospitals. Arrangements have been made, therefore, for conducting an ambulance service from this hospital direct to the field hospitals at the front and for sending attendants by train for patients who cannot be received directly by ambulance.

When patients are ready to return to their organizations, or are so nearly ready that it is necessary to wait for administrative reasons, it is very undesirable to have them transferred to an ordinary convalescent camp where they may succomb to the temptation of bringing to light again symptoms which had disappeared. Therefore Base Hospital 117 has been permitted to establish its own convalescent camp; discharge from which will be directly to the men's own organization.

The important modifications in administrative procedure that had to be made to enable division psychiatrists to carry on their work, and the special hospital at La Fauche to operate in the most efficient manner, have been due to the interest and insight into this very difficult new medico-military problem which have been shown by the Chief Surgeon of the American Expeditionary Forces. He has put the problem squarely up to those who presented the plans for its solution. With the cooperation of every medical officer we should attain a very considerable degree of success.

